# Development of a Predictive Model for Agave Prices Employing Environmental, Economic, and Social Factors: Towards a Planned Supply Chain for Agave-Tequila Industry

**DOI:** 10.3390/foods11081138

**Published:** 2022-04-14

**Authors:** Walter M. Warren-Vega, David E. Aguilar-Hernández, Ana I. Zárate-Guzmán, Armando Campos-Rodríguez, Luis A. Romero-Cano

**Affiliations:** 1 Grupo de Investigación en Materiales y Fenómenos de Superficie, Departamento de Ciencias Biotecnológicas y Ambientales, Universidad Autónoma de Guadalajara, Av. Patria 1201, Zapopan CP 45129, Jalisco, Mexico; wm.warren@edu.uag.mx (W.M.W.-V.); ana.zarate@edu.uag.mx (A.I.Z.-G.); luis.cano@edu.uag.mx (L.A.R.-C.); 2 Consejo Regulador del Tequila A. C., Av. Patria 723, Zapopan CP 45030, Jalisco, Mexico; daguilar@crt.org.mx; 3 Departamento de Ciencias Biotecnológicas y Ambientales, Universidad Autónoma de Guadalajara, Av. Patria 1201, Zapopan CP 45129, Jalisco, Mexico

**Keywords:** Tequila production, predictive model, agave planification and harvest, guarantee supply chain of agave-Tequila industry

## Abstract

The interest of consumers to acquire Tequila has caused an increase in its sales. As demand increases, the Tequila industry must obtain its raw material at a constant rate and agave farmers must be prepared to satisfy this supply chain. Because of this, modernization of the strategies used to ensure a planned, scheduled, timely, and predictable production will allow farmers to maintain the current demand for Tequila. This has been evidenced in official historical records from 1999 to 2020 where there is a fluctuation in the price of agave due to supply and demand. Given this scenario, this research shows the development of a multivariable predictive mathematical model that will permit the agave–Tequila production chain to work based on a smart implementation of planned actions to guarantee the agave supply to the Tequila industry. The proposed model has a goodness of fit (R = 0.8676; $\bar{R}$^2^ = 0.8609; F_(1,20)_ = 131.01 > F_0.01_ _(1,20)_ = 8.10) and demonstrates the impact on agave prices is due to several factors: Tequila exports (α = 0.50) > agave plants harvested “jima” (α = 0.44) > dollar exchange (α = 0.43) > Tequila production (α = 0.06) > annual accumulated precipitation (α = 0.05). Nevertheless, the price forecast can be influenced by climate change or economic crises that affect the supply chain. In conclusion, a prediction of agave price stabilization for five years is shown where authorized producers can evaluate future scenarios so that the agave supply chain can be guaranteed for Tequila production, facilitating the decision making regarding its raw material.

## 1. Introduction

**Figure 1:**
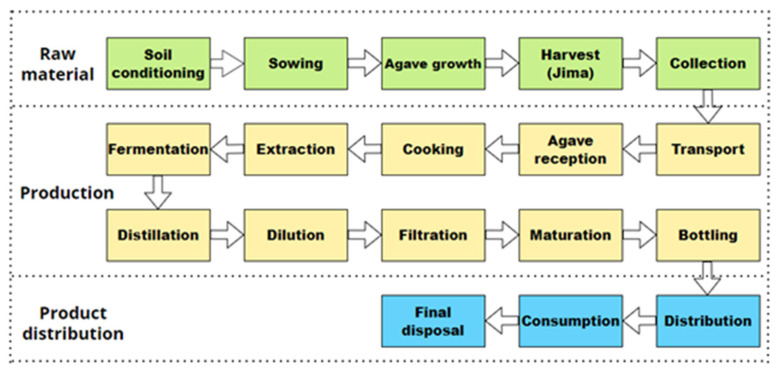
Supply chain of the agave–Tequila industry.

Tequila is a regional Mexican alcoholic beverage obtained by the distillation of musts, prepared directly and originally from the extracted material, in the companies of an authorized producer, which must be located within the territory that grants the Denomination of Origin of Tequila (DOT), derived from the heads of *Agave tequilana* Weber blue (a species that is characterized by being a succulent plant with a length of 1.2 to 1.8 m and lanceolate leaves of 90 to 120 cm with a bluish glaucous to grayish green color [1]), which undergo processes of hydrolysis, fermentation, and distillation. According to the historical data in 2021, Tequila and Tequila 100% agave categories had production increases of 10% and 60%, respectively, reaching a historical production record of 527.1 million liters, which demonstrates the positioning and economic strength of the industry in the different national and international markets. Therefore, supply chain assurance is no longer a matter of added value, but exists in a constant need of competitiveness and to assure the quality of the product. To maintain this constant growth rate in the Tequila industry, it is essential to secure the supply chain from start to finish. Figure 1 shows each of the stages that make up the productive chain of the agave–Tequila industry. The supply chain starts with the growth and harvest of the agave, which begins with the farmer ensuring that the development and growth of the agave is carried out in optimal conditions, thus avoiding phytosanitary problems such as pest infestation. Once it reaches an optimum size (from 6 to 8 years), the harvest, commonly known as “jima”, is carried out. For the “jima”, it is necessary that the farmer knows how to cut agave leaves and roots only collecting the *Agave tequilana* Weber blue variety “hearts” (pineapple without leaves and root). The next step is transportation, where agave hearts are transported as a raw material to the Tequila-producing companies with all the necessary documentation that demonstrates this agave has been certificated by the Tequila Regulatory Council. When the raw material arrives at the Tequila companies, several processes are carried out in a systematic way, ensuring at all moments the quality of the product in which masonry ovens, fermentation tanks and stills are always monitored. However, to ensure the supply chain is not affected, contingency plans are made to carry out preventive actions at the time that any stage in the production of the Tequila suffers an unforeseen event. This is achieved through an on-site verification within which inspectors must maintain a regular inspection routine. These quality controls are also carried out in the warehouse, as well as in the subsequent stages (bottling and packaging) with the purpose of guaranteeing safety to workers and the final product. The last stage of the supply chain is the transportation to its destination, where logistics plays an important role since the final product must be kept in an impeccable condition. Additionally, Tequila and its respective bottle must be maintained without suffering any damage that compromises the quality of the product and impacts on the image of the beverage. In summary, these can be grouped into three categories: (i) raw material, which is mainly the responsibility of the farmers; (ii) production, which is the responsibility of the Tequila production factories and covers all the operations for the transformation of the raw material to the final product; (iii) the distribution of the production and protection of the brand, which is the responsibility of the sales departments.

Among these stages, it has been highlighted that the first stage presents a great weakness due to cyclical shortage problems. Because of this, to ensure the constant growth of the industry, official regulations have been generated that protect farmers and producers, highlighting the Mexican standard that governs the DOT (NOM-006-SCFI-2012), which requires that agave plants used for Tequila production must have a plantation certificate that allows producer’s registration as an authorized producer under the responsibility of the Tequila Regulatory Council (CRT for its acronym in Spanish). Although these guarantees maintain the high-quality standards of the beverage, it has been shown that it does not fully protect the economy of the agave–Tequila production chain. Since the DOT region is an area with geographical limitations, the planting and harvesting of agave plants have the same limitations since the producers consider that they must be 7 or 8 years old to be harvested and used in the production of Tequila, because at this age the agave has the highest amount of total reducing sugars [2]. Although there is scientific evidence that the effect of agave age does not affect quality parameters of the beverage [3]. It has been appreciated that in the period from 1999 to 2020 there were registered fluctuations between the oversupply and scarcity of *Agave tequilana* Weber blue due to the time it takes for the plant to mature. The scarcity generates for the Tequila producers a shortage of raw material, high prices of the same, and the risk of not meeting production schedules and, consequently, consumers not receiving this beverage [2,4,5,6,7,8,9,10]. Therefore, agave producers can increase their profits due to there being low competition as there are few suppliers. On the other hand, the oversupply of agave plants guarantees the production schedules of Tequila companies, with the presence of the product in the market and low prices of the raw materials, but causing economic losses for agave producers, the abandonment of plantations, and social problems [6,7,11,12]. Both extreme sides cause a mismatch and damage to the production chain and its components, leading to a risk that the existing market demand for acquiring Tequila cannot be met. There have already been reported cases of the failure in taking preventive actions causing a decrease in sales of the product, thus affecting the image of the product. Such is the case of Bordeaux wine, in which agronomists did not have a tool that could prepare them to take action to satisfy the demand. Consequently, there were no profits, and the quality of the product decreased [13,14,15]. In addition, the evaluation of grapes belongs to the Rioja Qualified Designation of Origin (DOCa) in which determinants were needed to evaluate the feasibility of having transactions with wineries from this area [16]. For this reason, it is important to maintain a stable supply chain, within which none of its stages should be affected since these become subject to various political, economic, social, and even climatic events, which allows us to see the effect of demand, scarcity, and sales. This makes producers appreciate how the future of this industry is going and allows them to take timely actions that allows farmers and producers to be certain of the panoramas that are forecast with the current conditions [17]. In the literature, the use of predictive models has been reported to estimate the future prices of different raw materials both of animal and vegetable origin. Table 1 summarizes different models that have been used for price prediction. From this it can be identified that the increase in the prices of raw materials and products is a function of different variables that are characteristics of each product. Showing the compression between the relationship between the variables and the market prices of the products will allow the design of strategies to face the volatility of the market.

Due to the above, this work presents a descriptive investigation in which the variables that affect the change in price in the *Agave tequilana* Weber blue variety are analyzed. By studying the historical information, it will be possible to understand the phenomenon of the fluctuating change in its price, leading to it being possible to propose, as an alternative solution to the problem, a mathematical model capable of describing the historical data of the price of agave with a level of significance of 0.01. The proposed mathematical model will be able to forecast future prices of the raw material, which will be useful for farmers and Tequila producers to design strategic plans to ensure compliance with the Tequila supply chain to meet current demand.

**Table 1:** Summary of models for price prediction of raw materials and products.

Raw Material or Product	Model	Variables of the Model	References
Cereals	Spatial price prediction	Longitude, latitude, precipitation, month, and access to the market.	[18]
Corn	Nonlinear autoregressive models: univariate and bivariate neural network	Daily corn cash prices and future corn prices estimation.	[19]
	Multiple linear regression model	Production, import, outports, and consumption of corn.	[20]
Cotton	Multifactor seasonal model	Daily futures cotton prices	[21]
Soybeans	Multifactor seasonal model	Daily futures soybeans prices	[21]
	Quantile repression radial basis function (QR-RBF) neural network model	The output of domestic soybean, the import volume of soybean, the output of global soybean, the demand of domestic soybean, consumer price index, consumer confidence index, money supply, and port distribution price of imported soybean.	[22]
Olive oil	Autoregressive fractionally integrated moving average model (ARFIMA) and Fuzzy time series (FTS).	Consumption, import, export, and production.	[23]
Wheat	Radial basis function model (RBF).	Climatic and meteorological variables	[24]
Potato	Multivariate linear regression	Average temperature lowest temperature, daily temperature range, lowest grass temperature, relative humidity, lowest relative humidity, amount of cloud cover, solar radiation, sunshine, average wind velocity, amount of evaporation, ground-surface temperature.	[25]
Cocoa bean	Autoregressive integrated moving average (ARIMA) model.	Explanatory variables	[26]
Tomato	Seasonal ARIMA (SARIMA)	Weekly and monthly tomato market prices	[27]
	Backpropagation neural network (BPNN)	Weekly and monthly tomato market prices	[28]
	Backpropagation neural network (BPNN) and radial basis function neural network (RBF).	Weekly and monthly tomato market prices	[29]
Garlic	ARIMA-SVM hybrid model	Average monthly wholesale price of garlic	[30]

## 2. Materials and Methods

### 2.1. Data Analysis

Selection of the independent variables was conducted using the forward selection stepwise procedure [31]. It begins with no independent variables in the model, and successively adds variables to progressively establish a model of greater complexity where the addition of new variables ceases when there is no significant improvement in the predictions of the whole model.

The algorithm used to obtain the independent variable for this model can be summarized in two stages. At the initial step, the full set of candidate independent variables is reviewed, then the variable that has the most significant relationship to the dependent outcome variable is selected for inclusion in the model, as long as it complies with a certain minimum criterion level of significance. In the present work, this criterion was that the p-value for the variable to be included should be no greater than 0.01. The most significant variable (if any) that satisfies this criterion is then added to the model, and a solution to the functional form of the equation is computed. At the next stage, the remaining possible variables are considered in turn, to analyze if they significantly improve the prediction of the dependent variable, being conditional to the presence of the first variable already selected. As was previously mentioned, any variable to be included at this point must satisfy the significance probability criterion, but in this case, such probability is conditional on the presence of the first variable in the model. The most significant of these second step variables is then included into the model, and a new functional equation is computed. Finally, the set of variables remaining at each point is evaluated, where the most significant is included as long as it meets the criterion of statistical significance. The algorithm ceases to select further variables when no new significant variables can be identified.

The independent variables tested for the construction of the model were: (i) number of plants available, number of damaged plants, number of new plants planted (as supply indicators); (ii) total production of Tequila, the total export of Tequila, number of active Tequila factories (as supply demand); (iii) dollar exchange rate, Gross Domestic Product (GDP), cost of basic supplies for the management of agave nutrition (as an economic indicator); (iv) annual accumulated precipitation, night temperature, medium temperature (as weather indicator). Even though there exist other parameters that can influence agave prices such as counterfeit beverages, these practices must be considered totally illegal, and, through the support of Mexican authorities together with those of the different countries where Tequila is exported, quality controls have been reinforced. Thus, establishing agreements with various institutions for the detection and destruction of this fraudulent product, in addition to the CRT having a unit for final product assurance in which it is responsible for verifying that everything is kept in order and confiscating the adulterated product that affects the image of Tequila, is important as these products can affect the production chain. However, it is difficult to keep a detailed record of how much adulterated drink is confiscated during the year since it is destroyed before its commercialization. In the end, this is not related to production within the Tequila industry since it is under constant surveillance to maintain the quality criteria of excellence and provide the unique distinctiveness that Tequila gives to its consumers.

### 2.2. Information Regarding the Selected Independent Variables

As will be discussed later, the variables that most influence the price of agave are: (i) the number of plants available (supply indicator), (ii) total production of Tequila (demand indicator), (iii) Tequila exports (growth indicator), (iv) dollar exchange rate (economic indicator), and (v) annual accumulated precipitation (weather indicator). For this reason, this historical data were collected for the period from 1999 to 2020. The information was collected from official information sources that have rigorous procedures for its acquisition.

Response variable: Price of agave, MXN/ton. The historical data used for the price of agave were selected based on information reported by the government website of the Servicio de Información Agroalimentaria y Pesquera (SIAP). This government agency has a rigorous and strict system for obtaining information. The process begins with direct monthly visits to the farmers by highly trained technicians to request and record the information. After collecting the information, it is fed into their databases for later publication (Supplementary Table S1).

(I) Number of plants available, thousands of ton/year (supply indicator). The data used was retrieved from the databases of the Tequila Regulatory Council (CRT), which has a strict procedure used within this register; as a part of the completion of an application by the farmer for the registration of the cultivation field of new plants, they provide supporting documentation of the plantation that is under review (pre-registration). Afterwards, a field visit is carried out by the department in charge of the CRT, which oversees monitoring and verification that must be fulfilled in the field so that it can be found within the register of authorized producers. This evidence consists of counting the total number of plants, taking photographs of the field, and determining the geographical location of the property. Finally, to maintain a constant verification process, visits are scheduled in which the producer maintains a record of the partial and total sale of his plant inventory. (Supplementary Table S1).
(II) The total production of Tequila, millions of L/year (demand indicator) and the total export of Tequila, millions of L/year (growth indicator). The data of total production and export of Tequila were retrieved from public information that the CRT updates periodically. The collection of these data consists of a rigorous criterion in which weekly reports that each company registers and authorizes for Tequila production are submitted to the CRT. It is important to note that in addition to this, the CRT has a verification department responsible for carrying out unannounced audits to corroborate the information reported by each company and guarantee the quality in each one of the stages of the Tequila production process. (Supplementary Table S1).
(III) Dollar exchange rate, MXN (economy indicator). The dollar exchange rate data were retrieved from the official reports published by the Bank of Mexico (Banxico) through its Economic Information System website located within the exchange rate and historical results in the average exchange rate of Mexican pesos to United States of America dollar. (Supplementary Table S1).
(IV) Annual accumulated precipitation, mm (weather indicator). The annual accumulated precipitation data for the state of Jalisco, Mexico, were obtained from the databases of the National Water Commission (CONAGUA), an administrative agency whose structure is the National Meteorological Service. Historical data are expressed in “mm”, which corresponds to 1 L m^−2^. (Supplementary Table S1).

The compilation of the historical data used for each of the variables used in the development of the proposed mathematical model is presented in Supplementary Table S2.

### 2.3. Statistical Analysis: Multivariate Predictive Model

A nonlinear multivariable predictive model similar to that proposed by Peisheng et al. was used [32]. In our proposal, it is considered that the dependent variable “price of agave (y)” can be estimated based on 5 independent variables: “harvested plants, supply indicator (x_1_)”, “total Tequila production, demand indicator (x_2_)”, “average annual price of the dollar, economic indicator (x_3_)”, “liters of Tequila exported, growth indicator (x_4_)”, and “annual accumulated rainfall in the state of Jalisco, climatology indicator (x_5_)”.

The necessary assumptions for the model are: (i) Each value of $x_{i}$ and y are observed without measurement error; (ii) the relationships between y and each of the independent variables $x_{i}$ are linear in the parameters of the specific functional form of the model; (iii) each conditional distribution of $\mu_{i}$ has a mean of zero; (iv) the variance of the conditional distribution of $\mu_{i}$ is constant for all such distributions; (v) the values of $\mu_{i}$ are serially independent.

The proposed model has the following structure (Equation (1))

(1) $$\begin{array}{cc} y & =\mu_{01}+\mu_{02}+\mu_{03}+\mu_{04}+\mu_{05}+\beta_{11}x_{1}+\beta_{12}x_{2}+\beta_{13}x_{3}+\beta_{14}x_{4}\\ & +\;\beta_{15}x_{5}+\beta_{21}x_{1}^{2}+\beta_{22}x_{2}^{2}+\beta_{23}x_{3}^{2}+\beta_{24}x_{4}^{2}+\beta_{25}x_{5}^{2}\\ & +\;\beta_{31}x_{1}^{3}+\beta_{32}x_{2}^{3}+\beta_{33}x_{3}^{3}+\beta_{34}x_{4}^{3}+\beta_{35}x_{5}^{3}+\beta_{41}x_{1}^{4}\\ & +\;\beta_{42}x_{2}^{4}+\beta_{43}x_{3}^{4}+\beta_{44}x_{4}^{4}+\beta_{45}x_{5}^{4} \end{array}$$

where β_i1_ (i = 1, 2, 3, 4,) are the adjustment coefficients corresponding to the plants harvested that undergoes a process called “jima”, β_i2_ (i = 1, 2, 3, 4) correspond to total Tequila production, β_i3_ (i = 1, 2, 3, 4) to the dollar exchange rate, β_i4_ (i = 1, 2, 3, 4) correspond to Tequila exports, β_i5_ (i = 1, 2, 3, 4) for the annual accumulated precipitation of Jalisco state, and μ_0j_ (j = 1, 2, 3, 4, 5) correspond to the nonmeasurable uncertainties for each independent variable.

Additionally, it was decided to group the effect of each independent variable to place a weight parameter (α_i_) that determines the degree of effect that each independent variable has on the dependent variable, so the model can be rearranged in the following way (Equation (2))

(2) $$\begin{array}{cc} y & ={\;\;\;\alpha \;\;\;}_{1}\left({\;\;\;\mu \;\;\;}_{01}+{\;\;\;\beta \;\;\;}_{11}x_{1}+{\;\;\;\beta \;\;\;}_{21}x_{1}^{2}+{\;\;\;\beta \;\;\;}_{31}x_{1}^{3}+{\;\;\;\beta \;\;\;}_{41}x_{1}^{4}\right)\\ & +\;{\;\;\;\alpha \;\;\;}_{2}\left({\;\;\;\mu \;\;\;}_{02}+{\;\;\;\beta \;\;\;}_{12}x_{2}+{\;\;\;\beta \;\;\;}_{22}x_{2}^{2}+{\;\;\;\beta \;\;\;}_{32}x_{2}^{3}+{\;\;\;\beta \;\;\;}_{42}x_{2}^{4}\right)\\ & +\;{\;\;\;\alpha \;\;\;}_{3}\left({\;\;\;\mu \;\;\;}_{03}+{\;\;\;\beta \;\;\;}_{13}x_{3}+{\;\;\;\beta \;\;\;}_{23}x_{3}^{2}+{\;\;\;\beta \;\;\;}_{33}x_{3}^{3}+{\;\;\;\beta \;\;\;}_{43}x_{3}^{4}\right)\\ & +\;{\;\;\;\alpha \;\;\;}_{4}\left({\;\;\;\mu \;\;\;}_{04}+{\;\;\;\beta \;\;\;}_{14}x_{4}+{\;\;\;\beta \;\;\;}_{24}x_{4}^{2}+{\;\;\;\beta \;\;\;}_{34}x_{4}^{3}+{\;\;\;\beta \;\;\;}_{44}x_{4}^{4}\right)\\ & +\;{\;\;\;\alpha \;\;\;}_{5}\left({\;\;\;\mu \;\;\;}_{05}+{\;\;\;\beta \;\;\;}_{15}x_{5}+{\;\;\;\beta \;\;\;}_{25}x_{5}^{2}+{\;\;\;\beta \;\;\;}_{35}x_{5}^{3}+{\;\;\;\beta \;\;\;}_{45}x_{5}^{4}\right) \end{array}$$

using matrix operations that can be expressed as follows

$$y=\left[\begin{array}{ccc} {\;\;\;\alpha \;\;\;}_{1} & {\;\;\;\alpha \;\;\;}_{2} & \begin{array}{cc} {\;\;\;\alpha \;\;\;}_{3} & {\;\;\;\alpha \;\;\;}_{4} \end{array} \end{array}\right]\left[\begin{array}{cccc} x_{1} & x_{1}^{2} & x_{1}^{3} & x_{1}^{4}\\ x_{2} & x_{2}^{2} & x_{2}^{3} & x_{2}^{4}\\ x_{3} & x_{3}^{2} & x_{3}^{3} & x_{4}^{3}\\ x_{4} & x_{4}^{2} & x_{3}^{4} & x_{4}^{4}\\ x_{5} & x_{5}^{2} & x_{5}^{3} & x_{5}^{4} \end{array}\right]\left[\begin{array}{c} {\;\;\;\beta \;\;\;}_{1j}\\ {\;\;\;\beta \;\;\;}_{2j}\\ \begin{array}{c} {\;\;\;\beta \;\;\;}_{3j}\\ {\;\;\;\beta \;\;\;}_{4j} \end{array} \end{array}\right]+\left[\begin{array}{ccc} {\;\;\;\alpha \;\;\;}_{1} & {\;\;\;\alpha \;\;\;}_{2} & \begin{array}{cc} {\;\;\;\alpha \;\;\;}_{3} & {\;\;\;\alpha \;\;\;}_{4} \end{array} \end{array}\right]\left[\begin{array}{c} {\;\;\;\mu \;\;\;}_{01}\\ {\;\;\;\mu \;\;\;}_{02}\\ \begin{array}{c} {\;\;\;\mu \;\;\;}_{03}\\ {\;\;\;\mu \;\;\;}_{04} \end{array} \end{array}\right]$$

delimiting the set of matrices to

$$\;\;\;\alpha \;\;\;=\left[\begin{array}{ccc} {\;\;\;\alpha \;\;\;}_{1} & {\;\;\;\alpha \;\;\;}_{2} & \begin{array}{cc} {\;\;\;\alpha \;\;\;}_{3} & {\;\;\;\alpha \;\;\;}_{4} \end{array} \end{array}\right],\;f=\left[\begin{array}{cccc} x_{1} & x_{1}^{2} & x_{1}^{3} & x_{1}^{4}\\ x_{2} & x_{2}^{2} & x_{2}^{3} & x_{2}^{4}\\ x_{3} & x_{3}^{2} & x_{3}^{3} & x_{3}^{4}\\ x_{4} & x_{4}^{2} & x_{4}^{3} & x_{4}^{4}\\ x_{5} & x_{5}^{2} & x_{5}^{3} & x_{5}^{4} \end{array}\right],\;\;\;\;\;\;\;\;\;\;\;\beta \;\;\;=\left[\begin{array}{c} {\;\;\;\beta \;\;\;}_{1j}\\ {\;\;\;\beta \;\;\;}_{2j}\\ \begin{array}{c} {\;\;\;\beta \;\;\;}_{3j}\\ {\;\;\;\beta \;\;\;}_{4j} \end{array} \end{array}\right],\;\;\;\;\mu \;\;\;=\left[\begin{array}{c} {\;\;\;\mu \;\;\;}_{01}\\ {\;\;\;\mu \;\;\;}_{02}\\ \begin{array}{c} {\;\;\;\mu \;\;\;}_{03}\\ {\;\;\;\mu \;\;\;}_{04} \end{array} \end{array}\right]$$

where the simplified expression for the model can be defined as (Equation (3))

(3) $$y=\alpha \left(f\beta +\mu \right)$$

where f defines nonlinear functions that aggregate the effect of each independent variable on the dependent variable, and it is possible to define the independent variable with the most influence on the price of agave, the one that has the highest value of α_i_, and the variable with the least effect, the one with the lowest value of α_i_.

For the determination of the parametric adjustment, the method of least squares was used, the validity of the method has been corroborated by various investigations [32,33,34,35] and it consists of minimizing the sum of squares of the differences in the residuals between the known values of the dependent variable and those predicted by the model. The general idea is to relate the response of an output and based as a function of a vector of responses $X={\left[x_{1},x_{2},x_{3}\cdots x_{n}\right]}^{T}$ [36].

For the evaluation of the numerical values of the model, the MATLAB program version R2015a (MathWorks Inc, Apple Hill Drive, MA, USA) was used with the support of the multivariable nonlinear regression tool, which consists of constructing a mathematical function that best fits the data series with the possibility of being subject to value restrictions. Once the database was fed, the parameters were evaluated $\beta_{\mathrm{ij}}$ and $\mu_{j}$. Finally, with the support of the Solve tool, the matrix equations were solved to determine the values of the weight functions $\left(\alpha_{j}\right)$ to optimize the adjustment of the model.

### 2.4. Model Fit Quality Indicators

The statistical analysis of the proposed model was evaluated using the MINITAB 18 (Minitab Inc., State College, PA, USA) software for determination of the coefficient of determination (R), adjusted coefficient of determination (${\bar{R}}^{2}$), and the significance test of the regression model (F-test) [37,38]. The equations and methodology used are presented in detail in the Supplementary Material section.

## 3. Results and Discussion

### 3.1. Risk Analysis of the Supply Chain of the Tequila Industry to Satisfy Current Demand

**Figure 2:**
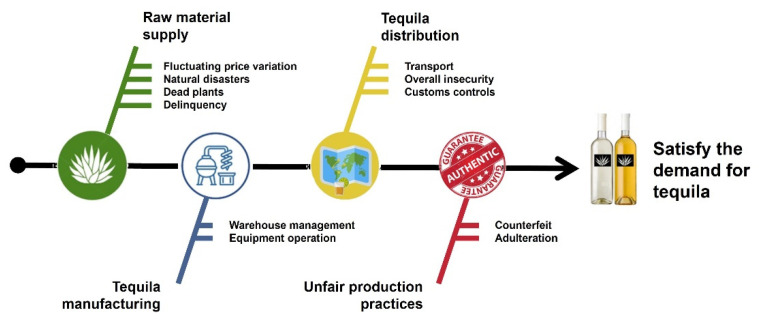
Risk analysis of the Tequila supply chain to satisfy the current demand.

According to 2021 data obtained from the Tequila Regulatory Council (CRT), the Tequila industry produced 352 million liters and exported to 120 countries, which demonstrates the success of the beverage among national and international consumers. Due to the success acquired, it must be taken into account that this demand will increase so it becomes a primary objective to ensure the ongoing supply chain of the product to maintain competitiveness (Figure 1). With the aim of satisfying the demand for the product, it is important to discuss those factors that will directly affect its fulfillment. From this perspective, the agave–Tequila production chain faces several challenges, shown graphically in Figure 2.

The first risk factor is the supply of raw materials for Tequila production (*Agave tequilana* Weber blue variety). Problems that directly affect this include: (i) the fluctuating variation in the price of agave [39], (ii) natural disasters that result in the loss of plants [40], (iii) the deaths of plants due to the spread of diseases [41,42,43].In addition, not only has there been a loss of plants caused by natural disasters, this has also been caused by criminal acts within the region, which can lead to periods of scarcity or price changes. Some examples have been the theft of plants that were in the cultivation fields as well as of the vehicles used for their transportation [44].

The second risk factor that affects the production chain is within the manufacturing process of the beverage and is related to problems caused by poor operating practices due to inefficient equipment and poor stock management, which can cause slower response times. In this sense, the industry has begun to update and professionalize itself by following good production practices, redesigning equipment, and innovating in key stages of the production process, such as hydrolysis, fermentation, distillation, and maturation [3,45,46,47,48,49].

Another risk factor for satisfying the current demand is the distribution of the final product since it has been shown that transport and logistics problems can be decisive in the sale of the product, as well as problems associated with customs controls that can delay the distribution of the product. In this sense, there are current practices aimed at distributing the product at a national and international level that are working successfully, so that the product can be available in 120 countries. In some countries, such as Mexico, the distribution can be carried out by the same production company; however, in large markets, it is necessary to hire regional distributors who are in charge of carrying out this task, such as in the case of the United States of America [50,51].

Finally, it is important to highlight a fourth risk factor caused by the unfair practices of some producers whose product is counterfeit or adulterated, as has happened with other successful alcoholic beverages [52,53]. According to recent studies, it has been shown that the realization of these illegal practices has been one of the greatest obstacles to impact the supply chain of alcoholic beverages because they can cause damage to the health of the consumer, in addition to impacting the product’s image, which has an impact on the economy of the beverage [54,55]. In order to be able to stop these practices and be able to safeguard the image of Tequila and all the processes that compose it, the Tequila industry has a permanent inspection process carried out by the Tequila Regulatory Council (CRT) that allows monitoring of the quality and authenticity of the raw material and the final product. Although the results now are satisfactory, these procedures could be complemented with current methodologies such as those demonstrated by Gayialis et al. (2021) who have proposed the application of new technologies to generate anti-counterfeiting measures such as labels and smart sensors to offer effective traceability at all stages of the wine and spirits supply chain [56].

Due to the increasing demand for Tequila, the incidence of these practices has been constantly increasing, so quality and authenticity controls of the product have needed to evolve to take quick actions to accept or reject batches and achieve their distribution. Among the analytical techniques presented in the literature, the use of gas chromatography coupled to mass spectrometry of isotope ratios stands out since it has been shown that it is possible to determine robust analytical parameters, which can be useful as an auxiliary criterion for the processes of current verification [57,58,59].

Based on the above information, it is possible to conclude that for the risks of the production chain related to the manufacture of Tequila, the product’s distribution, and practices of counterfeiting/adulteration of the product, strategies already exist in order to ensure that the demand for the product is satisfied. However, the risk factor associated with the supply of raw material still needs to be studied and strategies proposed to achieve compliance to strengthen the Tequila supply chain.

### 3.2. Information Description: Risk Factor Analysis “Raw Material Supply”

**Figure 3:**
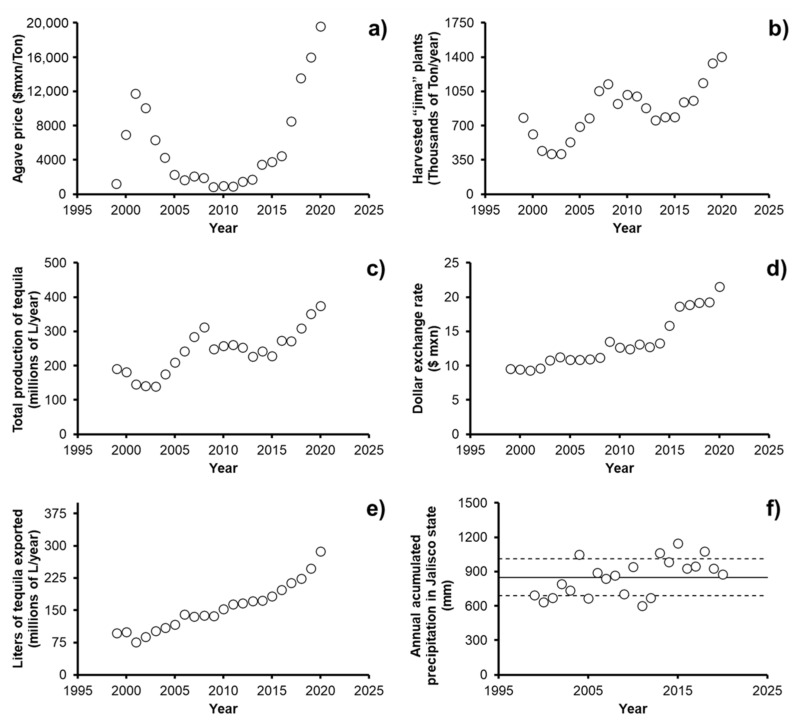
Historic data of: (**a**) agave price, (**b**) harvested “jima” plants, (**c**) Tequila total liters production, (**d**) dollar exchange rate, (**e**) Tequila exports, and (**f**) annual accumulated precipitation of Jalisco state, Mexico.

The data obtained for the change in the: price of agave, harvested “*jima*” plants, Tequila production, dollar exchange price, Tequila exports, and annual accumulated precipitation (from Jalisco state), with respect to the time in the period 1999 to 2020 are shown in Figure 3. When analyzing the fluctuations in the price of agave (Figure 3a), four clearly marked behaviors are observed: (i) an increase in the period 1999 to 2001, (ii) a decrease in the period 2002 to 2005, (iii) a period of stability from 2006 to 2013, and (iv) an exponential increase from 2014 to 2020.

The first price rise (1999 to 2001) can be associated with the combination of two factors. Firstly, the natural behavior of the market meant that as there was an increase in the demand for Tequila in international markets in this period (Figure 3e), the cost of agave became more expensive due to the need to satisfy the markets. The second factor is associated with the death of plants due to climate change during that period. This factor can be represented with the accumulated precipitation data in the Jalisco region (Figure 3f). It is observed that for these years the factor is out of control (below one standard deviation, dashed line) with values very close to 600 mm, although the water requirements of the *Agave tequilana* Weber blue variety to guarantee a good yield require plantation areas to have rainfall of between 600 and 1500 mm. Additionally, as a consequence of the climatic changes in this period, night temperatures below 11 °C were also recorded in the region, causing frost and damage to the development of the agave because it is a plant with a CAM-type photosynthesis process, with stomata closed during the day and open at night.

During the second period (2002 to 2005), there is an abrupt decrease in agave prices, which can be attributed to a decrease in harvested plants (Figure 3b) due to the low demand for total Tequila production (Figure 3c) and exports (Figure 3d). This occurred because the high costs of the raw material presented in the previous period forced the final product to become more expensive, resulting in a decrease in its national consumption. Given this scenario, the opening of international markets began, mainly in the United States and Europe, to offer the product to consumers with greater purchasing power.

In the case of the harvested plants, this variable mainly had an impact on the Tequila production process since the availability or absence of the raw materials impacted the price offered. In the 2009–2010 period, there was an increase of 91 thousand tons with a subsequent decrease in the 2011–2015 period, to finally recover in 2016 and continue to increase until today. Within this period, the presence of diseases and pests that affect the physical integrity of the *Agave tequilana* Weber blue variety plant were reported, which can be associated with drastic climate changes due to global warming. These phytosanitary problems caused, in 2010, 40 million plants to be lost, with an economic repercussion of 1635 million pesos. Due to this, campaigns were established in 2013 for the protection of the agave to promote and eliminate pests that affect the health of the plant [60]. It is estimated that if these actions are not carried out, the economic losses could reach 3700 million pesos [61]. Therefore, within the 2013–2015 period, all possible measures were carried out to reduce the infestation points and, thus, the infected fields with these pests, which was reflected in 2016 with an increase in the number of plants and, with this, in Tequila production.

The period of stability in the price of agave (2006 to 2013) is attributed to the fact that in that period, exports remained stable, demonstrating an exponential increase until 2014 due to the opening of markets [62]. One of these events was the opening and the growing interest that China had regarding the commercialization of Tequila within its territory, thus leading to an increase in sales of the Mexican beverage in China [63]. In addition to this, the dollar exchange rate (Figure 3d) remained without significant changes, except for the years 2008 to 2010 where the increase from MXN 11 to MXN 13 can be associated with the small increase in the price of agave registered in the period. The foregoing information was due to the problems caused by the world economic crisis that began in 2008 in the United States attributable to failures in economic regulation, the overvaluation of financial products, world food crisis, rise in oil prices due to the armed conflicts in Asia, and the credit/mortgage crisis [64]. This economic indicator directly impacts the acquisition of agricultural products such as fertilizers and insecticides that represent a necessary expense to achieve greater crop protection [65].

**Figure 4:**
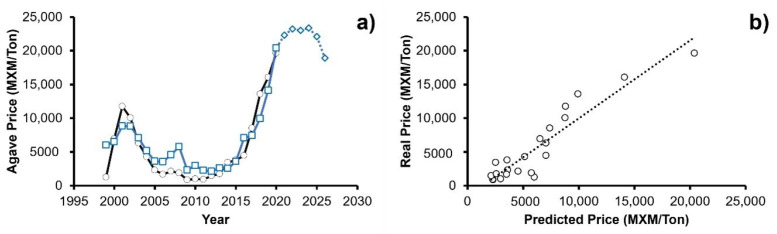
(**a**) ○ Historical data of the price of agave, □ predicted data of the predictive mathematical model for agave price, ◊ estimated projection for the next 6 years using the predictive mathematical model; (**b**) Comparison between the historical data vs. predicted data from the predictive mathematical model proposed.

Finally, in the current stage, an exponential growth in the price of agave (2014 to date) can be seen, which is attributed to the combination of the five factors studied: (i) the rise in exports with an exponential increase due to the opening of new markets (Figure 4); consequently, (ii) harvested plants and (iii) Tequila production show the same behavior; (iv) the dollar exchange rate is on the rise with historical records, mainly due to the change in the Mexican government and the economic crisis caused by the spread of COVID-19; (v) erratic climate changes due to global warming.

### 3.3. Predictive Model Proposal

Once the previous information was analyzed, the adjustment of the historical data to the proposed model was tested. The values for each of the evaluated parameters are shown in Table 2, while the graphic description of the model is shown in Figure 4a.

### 3.4. Fit Quality of the Model

In order to evaluate the goodness of fit and significance of the model, the R, ${\bar{R}}^{2}$, and F statistics were obtained using Equations (1)–(3). OriginPro 8.5.0 SR1 (Origin Lab., Northampton, MA, USA) statistical software was used for calculations. For all determinations, a significance level of 1% was used. Table 3 shows the values of agave price history statistics and model fitting for 1999 to 2020.

R = 0.8676; R^2^ = 0.8609; F_(1,20)_ = 131.01 > F_0.01 (1,20)_ = 8.10

For the correlation coefficients, R and ${\bar{R}}^{2}$ values of 0.8676 and 0.8609 were obtained, respectively, which present a minimum difference between them. In turn, the value of F was contrasted with the critical values of the F distribution reported in the literature [66]. Considering a significance level of 1%, the degrees of freedom of the regression variation, and the degrees of freedom of the residual variation, the critical value of F_0.01(1,20)_ = 8.10. For the present model, a value of F = 131.01 was obtained, therefore, F_model_ > F_0.01(1,20)_. This implies that the regression equation is meaningful and adequately represents the real prices for agave.

**Table 3:** Agave price 1999–2020 history statistics and model fitting.

Year	Real Price MNX/Ton	Predicted Price MNX/Ton	Residuals $(e=Y-\hat{Y)}$	Standardized Residuals $(e/\sqrt{MSE)}$
1999	1232.96	6196.49	2321.03	1.47
2000	6926.64	6667.79	−1353.10	−0.86
2001	11,731.55	9071.88	−2447.33	−1.55
2002	10,039.68	9492.86	−794.54	−0.50
2003	6310.24	7766.85	194.76	0.12
2004	4277.80	6562.61	470.28	0.30
2005	2295.51	4485.90	−163.19	−0.10
2006	1681.55	4828.56	626.48	0.40
2007	2121.12	5724.58	1202.47	0.76
2008	1886.75	6693.65	2342.18	1.48
2009	858.19	3220.42	−382.19	−0.24
2010	979.68	4531.76	840.70	0.53
2011	913.63	2678.00	−964.97	−0.61
2012	1470.03	2809.74	−1238.33	−0.78
2013	1754.47	3961.62	−293.54	−0.19
2014	3447.14	3926.71	−1560.84	−0.99
2015	3778.54	4618.95	−1109.89	−0.70
2016	4470.78	7810.69	1577.86	1.00
2017	8518.73	8179.13	−1000.91	−0.63
2018	13,563.44	10,567.02	−2285.93	−1.45
2019	16,039.45	15,148.60	492.93	0.31
2020	19,618.31	20,787.43	3526.09	2.23

It is concluded that the proposed model shows a good ability to predict the price of agave. Figure 4b compares the historical price of the agave and the prediction of the model in the same period, corroborating the validity of the prediction (R = 0.86).

When analyzing the function of weight (parameter α), it can be concluded that the variable that most affects the price of agave is the liters of Tequila exported (α_3_ = 0.50), this being dependent on the harvested plants (α_1_ = 0.44) and total production of Tequila (α_2_ = 0.05). As mentioned before, as there is a greater demand for the beverage, the cost of the raw material increases; however, it is important to note that this exponential growth is about to disappear. The proposed predictive model forecasts a period of stability in prices for the next 5 years and a subsequent fall, which is associated with the fact that the Tequila industry has already reached the opening of most international markets. Consequently, the demand for Tequila should remain the same or show slight increases due to advertising campaigns to encourage its consumption.

**Figure 5:**
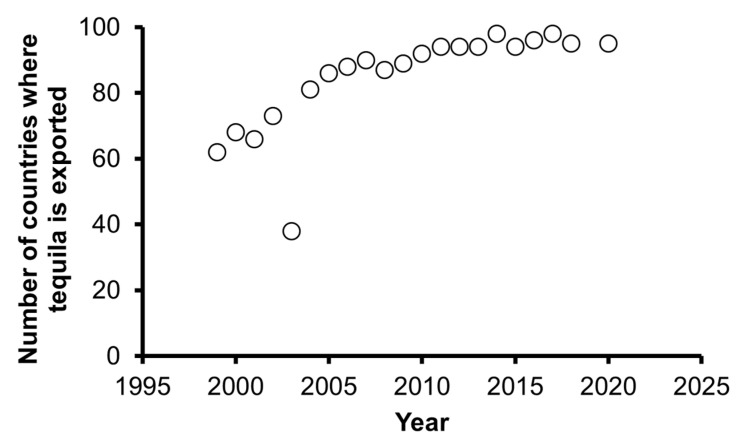
Historical data of the opening of international markets for Tequila commercialization.

This is evidenced by the information in Figure 5 and Table 4. Figure 5 shows the total number of countries to which Tequila was exported in the period 1999 to 2020. As of 2015, stability has been achieved in the opening of new markets, achieving the presence of the Tequila brand in 95 countries in 2020.

Finally, the weight functions for the dollar exchange (α_3_ = 0.43) and accumulated annual precipitation (α_5_ = 0.05) are shown as factors that affect the price of agave, but whose impact is less compared to the other factors. It is important to note that changes in these two variables will depend on external factors, as discussed previously: economic crises caused by public health, war, economic, and climatic problems. It is estimated that these two variables will be the ones that dictate the future behavior of the price of agave if exports remain without significant changes.

Table 2 shows the distribution of liters of Tequila exported in 2020 to the different continents. Currently, the commitment to maintain and boost the growth in demand for the beverage has focused on the Asian and Oceanian markets, with Australia, Japan, China, Turkey, and Russia being the countries with the highest consumption in the region. The strategy used to promote the brand in these regions should be the promotion of programs to spread the culture of Tequila through advertising campaigns and scientific dissemination programs by the CRT to show the high standards of quality and authenticity of the beverage.

### 3.5. Proposals for a Smart Agronomic Production

Using the predictive model, a future estimate of the price of the agave was made for the next 6 years (average time from when the agave is cultivated until its harvest) to provide information to the farmer and help in the strategic planting of the crop to increase its benefits in their productivity. This information is also useful for Tequila companies since it allows them to plan the acquisition of raw materials, increasing the benefit for all parties involved. It is forecast that the current demand for agave will remain in the following years, so planned actions must be taken to satisfy current markets.

One of the proposals that has gained great acceptance among Tequila producers is the use of young agave plants (4 to 6 years old) in such a way that cultivation and harvest times are reduced, managing to regulate cyclical changes in the price of the plant due to the time it takes to mature. Although there are alterations in the concentration of higher alcohols after fermentation due to its use, the distillation stages rectify the final product in such a way that this action does not compromise the parameters of quality and authenticity of the beverage [3].

Another of the planned actions is the realization of state and federal agreements that seek to protect the cultivation fields of agave in the region granted by the DOT. As already discussed, global warming affects climatic conditions, resulting in abrupt and sudden changes caused by erratic temperatures and rainfall, as well as the spread of diseases that affect the health of plantations. Agreements must be established in which campaigns and phytosanitary measures are put in place to provide a control, and where appropriate, eradication of and/or reduction in the damage caused by diseases associated with climate change. This can be similar to the agreements established to achieve the eradication of diseases caused by *Scyphophorus acupunctatus*, *Fusarium moniliforme*, *Fusarium oxysporum*, *Thielaviopsis paradoxa*, *Cercospora agavícola*, and/or *Pectobacterium carotovora*, which act alone or in combination to reduce plant resistance and also the quantity and quality of agave hearts “pineapple” production, impacting the increase in production costs.

**Figure 6:**
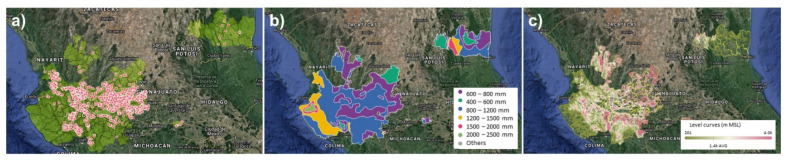
Maps corresponding to the region granted by the DOT: (**a**) geographical location of properties where *Agave tequilana* Weber blue variety jima was grown in the year 2020; (**b**) accumulated annual precipitation contour map; (**c**) altitude contour map with respect to sea level.

Figure 6a presents the geographical distribution of farms where *Agave tequilana* Weber blue variety “jima” was carried out in 2020. One of the planning actions could be the spread of cultivating agave plants in new areas; however, the farmer must be aware of the specifications for the adaptation of this plant because its conditions of adaptation mean that it is better grown in semi-arid and sub-humid regions (Figure 6b). It shows a low tolerance to low temperatures and prefers to be grown in medium-textured soils such as clay loam, or loam-sandy, on terrain with a high degree of slope (Figure 6c). Therefore, the geoclimatic situation is an essential variable to consider and undoubtedly impacts the price of agave [67].

Another aspect to consider is the implementation of innovative technologies that allow efficiency to be improved within the stages of Tequila production, from the agricultural sector to the industrial sector. As the industry provides more efficient processes, a smaller number of agave plants will be required to satisfy demand, thus reducing the prices of the raw material. Within this, various solutions have been proposed to address the issue, such as the development of equipment that allows improvement in the efficiency of the grinding and hydrolysis processes, such is the case with automatic rippers and diffusers. In addition, the automation of key stages in the process, such as in the distillation columns, would lead to a reduction in human error. This has already been put in place in several Tequila companies, and the advantages of automation have been shown in terms of increased efficiency, cost reduction, and improvements in safety and quality within the production stages [45,68,69,70,71,72].

Finally, Del Real Laborde [73] has proposed a planification to control the supply chain with two strategies: one based on the Tequila producer controlling production entirely and the other based on the speculative purchase of raw material available on the free market. Between these two extremes, there will be a multitude of combinations between the two links of the chain. This communication presents as a planning strategy for the management of the market in an organized way, making a predictive mathematical model available to the scientific and technological community; in addition, DOT maps from the areas where the research has been carried out are highlighted. Figure 6 shows the agave harvest in the year 2020 (Figure 6a), the contour curves that delimit the areas with average annual precipitation (Figure 6b), and the contour lines that demarcate the altitudes of the region (Figure 6c). These serve the purpose of ensuring agave producers know the potential market to which they will offer their production, and a Tequila producer can develop a plan for their future market based on the availability and costs involved with the supply strategy that it decides to follow. It is important to point out that the information in Figure 6, as well as the historical reports, are annually updated by the Tequila Regulatory Council and are available on its website (Supplementary Table S1). The information and scientific development shown in this research work opens the door to developing an informed market that allows future actions to be taken to satisfy needs.

## 4. Conclusions

It is possible to use the historical data regarding the price of agave to determine a predictive mathematical model that allows the prediction of future scenarios, which would be useful as a reference to develop strategic planning within the agave–Tequila industry supply and production chain, thus allowing this industry to maintain its constant growth rate and assure the quality of the product. The information presented here will allow minimization of the abrupt cyclical changes that prices have demonstrated in the last 30 years, increasing the benefits for all participants. The selection of the variables that have the greatest influence on determining the price of agave is appropriate since the model shows a good fit (R = 0.8676; $\bar{R}$^2^ = 0.8609; F_(1,20)_ = 131.01 > F_0_._01 (1,20)_ = 8.10), which gives a good future price prediction. A period of five years of stability of the price of agave is estimated because the Tequila brand finds stability in the international market with participation in 97 countries. Fluctuations that the price may suffer from can be associated with shortages due to phenomena that may occur due to climate change or economic crises that impact the supply chain. It is important to note that the predicted price has the limitation of being an estimation and should not be considered invariable for any reason since the variables on which it depends are associated with economic and social factors that can be altered by unforeseen events. Likewise, the databases used to generate the model and the contour graphs presented in this research must be continuously updated to guarantee an adequate prediction.

Finally, it is important to highlight the perspectives of the research. Although the proposed variables satisfactorily predict fluctuations in the price of *Agave tequilana* Weber blue variety, these are not the only factors that directly affect it. Criminal acts associated with the theft of plants are a key factor that must be studied further to obtain reliable data that can be entered into the model. A key step in obtaining this information is the use of drones to monitor agave crops. Likewise, the monitoring of key factors for plant growth such as night weather conditions and the presence of diseases must be monitored regularly to have historical data that allow the prediction of future scenarios. In addition, it is proposed to evaluate historical data on the growth of sales of other alcoholic beverages produced from other varieties of agave (mezcal, sotol, raicilla, and bacanora) since in recent years, these have gained acceptance in the market, which has repercussions as they may be direct competition for Tequila.

## Figures and Tables

**Table 2:** Parameters of the predictive mathematical model proposed for determining the price of agave based on: $\beta_{i1}\left(i=1,2,3,4,\right)$ number of plants available; $\beta_{i2}\left(i=1,2,3,4\right)$ production of Tequila; $\beta_{i3}\left(i=1,2,3,4\right)$ dollar exchange rate; $\beta_{i4}\left(i=1,2,3,4\right)$ total export of Tequila; $\beta_{i5}\left(i=1,2,3,4\right)$ annual accumulated precipitation; $\mu_{j}$ $\left(j=1,2,3,4,5\right)$ unmeasurable uncertainties and weight parameters $\alpha_{j}\;\left(j=1,2,3,4,5\right)$.

Parameters for the Predictive Mathematical Model					
Β_ij_	j = 1	j = 2	j = 3	j = 4	j = 5
i = 1	103.30	495.10	−4.17 × 10^4^	247.70	−138.00
i = 2	−0.26	−4.93	3526.00	−0.33	−3.49
i = 3	2.22 × 10^−4^	0.02	−133.90	2.07 × 10^−4^	0.03
i = 4	5.62 × 10^−8^	1.58 × 10^−5^	2.03	5.08 × 10^−8^	5.13 × 10^−5^
Parameter μ_j_					
	−2745.00	−969.70	1.83 × 10^5^	−6.57 × 10^4^	3.15 × 10^4^
Parameter α_j_					
	0.44	0.05	0.45	0.50	0.05

**Table 4:** Tequila sales participation percentage in the international markets from 2020.

Continent	Countries	Liters of Tequila Exports from 2020	Participation (%) in the Market
America	31	301,367,647.44	89.5
Europe	32	24,662,303.48	7.3
Asia	25	6,032,626.59	1.8
Africa	7	1,619,565.33	0.5
Oceania	2	3,217,762.82	1.0
Total	97	336,899,905.66	100.0

## Data Availability

The data that support the findings of this study are available from the corresponding author upon reasonable request.

## Supplementary Materials

The following supporting information can be downloaded at: https://www.mdpi.com/article/10.3390/foods11081138/s1, Table S1: Access link to the databases of the model variables., Table S2: Data of the selected independent variables
