# Supplementary material for: Development of a Predictive Model for Agave Prices Employing Environmental, Economic, and Social Factors: Towards a Planned Supply Chain for Agave-Tequila Industry
Source: Foods. 2022 Apr 14;11(8):1138. doi: 10.3390/foods11081138 (PMC9028388; doi:10.3390/foods11081138)
Supplement: Supplementary file 1 [file foods-11-01138-s001.zip › foods-1623267-supplementary.pdf]

foods

MDPI

Article

Development of a Predictive Model for Agave Prices Employing Environmental, Economic, and Social Factors: Towards a Planned Supply Chain for Agave-Tequila Industry

Walter M. Warren-Vega [1,†], David E. Aguilar-Hernández [2,3], Ana I. Zárate-Guzmán [1,‡], Armando Campos-Rodríguez [3,*] and Luis A. Romero-Cano [1]

[1] Grupo de Investigación en Materiales y Fenómenos de Superficie, Departamento de Ciencias Biotecnológicas y Ambientales, Universidad Autónoma de Guadalajara, Av. Patria 1201, Zapopan CP 45129, Jalisco, Mexico; wm.warren@edu.uag.mx (W.M.W.-V.); ana.zarate@edu.uag.mx (A.I.Z.-G.); luis.cano@edu.uag.mx (L.A.R.-C.)
[2] Consejo Regulador del Tequila A. C., Av. Patria 723, Zapopan CP 45030, Jalisco, Mexico; daguilar@crt.org.mx
[3] Departamento de Ciencias Biotecnológicas y Ambientales, Universidad Autónoma de Guadalajara, Av. Patria 1201, Zapopan CP 45129, Jalisco, Mexico
* Correspondence: armando.crodriguez@edu.uag.mx
† Current address: Centro de Investigación y Desarrollo Tecnológico en Electroquímica (CIDETEQ), Parque Tecnológico Sanfandila, Pedro Escobedo CP 76703, Querétaro, Mexico.
‡ Current address: Centro de Investigación y Estudios de Posgrado, Facultad de Ciencias Químicas, Universidad Autónoma de San Luis Potosí, San Luis Potosí CP 78060, Querétaro, Mexico.

Supplementary Materials

Model fit quality indicators

Comparison of observed and predicated data is typically used to judge the ability of models to adequately represent the process of interest. Some of the goodness-of-fit tests applied to multivariate regression models are based on the calculation of statistics such as the coefficient of determination (Equation S1), adjusted coefficient of determination (Equation S2), and the significance test of the regression model (F-test) (Equation S3).

$$R = \sqrt{1 - \frac{\sum\left(Y_i - \hat{Y}_i\right)^2}{\sum(Y_i - \bar{Y})^2}} \qquad \text{(Equation S1)}$$

Where:

$$\hat{Y}_i = Predicted\ values\ from\ the\ model$$

$$\bar{Y} = \frac{1}{n}\sum Y_i$$

However, the coefficient of determination (R) has the limitation that its value increases and approaches one the more variables are included in the model, which is an undesirable characteristic. In an attempt to solve this problem, a correction to the degrees of freedom is applied to transform the value of R into an adjusted coefficient of determination $\bar{R}^2$ [37].

$$\bar{R}^2 = 1 - \frac{\frac{\sum(Y_i - Y_i)^2}{df\ error}}{\frac{\sum(Y_i - \bar{Y})^2}{df\ error}} \qquad \text{(Equation S2)}$$

2.3.1. Significance Test of Regression Model (F-Test)

F test is an estrict evaluation for regression equation, where the supposition is

$$H_0: \beta_1 = \beta_2 = \cdots = \beta_m = 0$$

If H_0 is accepted, then the relationship of the proposed model and the independent variables $x_1, x_2, \ldots, x_m$ it is not significant.

F test can be expressed as follows:

$$F = \frac{\sum\left(\hat{Y}_i - \bar{Y}\right)^2 / (m-1)}{\sum\left(Y_i - \hat{Y}_i\right)^2 / (n-m)} \sim F(m-a, n-m) \qquad \text{(Equation S3)}$$

Where $m\text{-}1$ are the degrees of freedom of the regression variation and $n\text{-}m$ represents the degrees of freedom of the residual variation.

If $F > F_{(m-1,\, n-m)}$ then it is considered that there is a significant relationship between the variable y and the independent variables $x_1, x_2, \ldots x_m$ under a level of significance [38].

All statistical analyzes described in section 2.3 were performed using Minitab 18 software.

Table S1. Access link to the databases of the model variables.

Variable	Meaning	Link to the database of the data
y	Price of agave	https://www.gob.mx/siap/documentos/siacon-ng-161430
x_1	Number of plants available	https://www.crt.org.mx/EstadisticasCRTweb/
x_2	The total production of tequila	https://www.crt.org.mx/EstadisticasCRTweb/
x_3	Dollar exchange rate	https://www.banxico.org.mx/SieInternet/
x_4	The total export of tequila	https://www.crt.org.mx/EstadisticasCRTweb/
x_5	Annual accumulation precipitation	https://smn.conagua.gob.mx/es/climatologia/temperaturas-y-llu-vias/mapas-diarios-de-temperatura-y-lluvia

Table S2. Data of the selected independent variables.

Data of the selected independent variables					
**Year**	**Plants available (thousands of Ton/year)**	**Tequila production (millions of L/year)**	**Dollar exchange rate ($ mxn)**	**the total export of tequila (millions of L/year)**	**Annual precipitation (mm)**
1999	780	191	10	97	692
2000	615	182	9	99	632
2001	443	147	9	76	671
2002	414	141	10	88	792
2003	413	140	11	102	736
2004	530	176	11	109	1050
2005	689	210	11	117	664
2006	779	243	11	140	892
2007	1054	284	11	135	837
2008	1125	312	11	137	869
2009	925	249	14	136	704
2010	1015	258	13	153	940
2011	998	261	12	164	602
2012	881	253	13	167	669
2013	757	227	13	172	1063
2014	788	242	13	173	982
2015	789	229	16	183	1146
2016	942	273	19	198	926
2017	956	271	19	213	946
2018	1139	309	19	224	1076
2019	1343	352	19	247	926
2020	1407	374	21	287	874
